# Decreased Serum Hepcidin Concentration Correlates with Brain Iron Deposition in Patients with HBV-Related Cirrhosis

**DOI:** 10.1371/journal.pone.0065551

**Published:** 2013-06-11

**Authors:** Dong Lin, Jing Ding, Jian-Ying Liu, Yi-Feng He, Zhi Dai, Cai-Zhong Chen, Wei-Zhong Cheng, Jian Zhou, Xin Wang

**Affiliations:** 1 Department of Neurology, Zhongshan Hospital, Fudan University, Shanghai, China; 2 Institute of Brain Science and State Key Laboratory of Medical Neurobiology, Shanghai, China; 3 Liver Cancer Institute, Zhongshan Hospital, Fudan University, Shanghai, China; 4 Department of Radiology, Zhongshan Hospital, Fudan University, Shanghai, China; 5 Shanghai Key Laboratory of Organ Transplantation, Shanghai, China; Queen Mary University of London, United Kingdom

## Abstract

**Purpose:**

Excessive brain iron accumulation contributes to cognitive impairments in hepatitis B virus (HBV)-related cirrhotic patients. The underlying mechanism remains unclear. Hepcidin, a liver-produced, 25-aminoacid peptide, is the major regulator of systemic iron metabolism. Abnormal hepcidin level is a key factor in some body iron accumulation or deficiency disorders, especially in those associated with liver diseases. Our study was aimed to explore the relationship between brain iron content in patients with HBV-related cirrhosis and serum hepcidin level.

**Methods:**

Seventy HBV-related cirrhotic patients and forty age- sex-matched healthy controls were enrolled. Brain iron content was quantified by susceptibility weighted phase imaging technique. Serum hepcidin as well as serum iron, serum transferrin, ferritin, soluble transferrin receptor, total iron binding capacity, and transferrin saturation were tested in thirty cirrhotic patients and nineteen healthy controls. Pearson correlation analysis was performed to investigate correlation between brain iron concentrations and serum hepcidin, or other iron parameters.

**Results:**

Cirrhotic patients had increased brain iron accumulation compared to controls in the left red nuclear, the bilateral substantia nigra, the bilateral thalamus, the right caudate, and the right putamen. Cirrhotic patients had significantly decreased serum hepcidin concentration, as well as lower serum transferring level, lower total iron binding capacity and higher transferrin saturation, compared to controls. Serum hepcidin level negatively correlated with the iron content in the right caudate, while serum ferritin level positively correlated with the iron content in the bilateral putamen in cirrhotic patients.

**Conclusions:**

Decreased serum hepcidin level correlated with excessive iron accumulation in the basal ganglia in HBV-related cirrhotic patients. Our results indicated that systemic iron overload underlined regional brain iron repletion. Serum hepcidin may be a clinical biomarker for brain iron deposition in cirrhotic patients, which may have therapeutic potential.

## Introduction

Patients with hepatic cirrhosis exhibit a spectrum of neurologic complications, ranging from profound neurological dysfunction to mild neuropsychiatric deficits [1], [2], [3]. Burkhard *et al* found 21.6% patients exhibited definite Parkinsonism in a one-year follow-up study [4]. Jalan *et al* found about 67% cirrhotic patients showed evidence of cerebral dysfunction in neuropsychological tests [5]. Naik *et al* found even patients with Child’s A liver function presented subclincial cognitive alterations which could make patients impossible to perform normal daily tasks [6], [7]. Decreased essential substances for normal brain function, or increased toxic metabolites in the brain may underlie the pathogenesis of neurological complications in liver dysfunction. These toxic substances included ammonia, GABA, and heavy metals [1], [8].

Iron, an essential paramagnetic ion, is found in the systemic organs and the central nerve system [9]. Excessive brain iron deposition was found in Parkinson’s disease [10], Alzheimer’s disease [11], multiple sclerosis [12], Friedreich’s ataxias [13], neuroferritinopathy [14], and Hallervorden-Spatz syndrome [15]. Our previous study revealed that excessive iron accumulation in the frontal cortical-basal ganglial circuit independently contributed to cognitive impairments in minimal hepatic encephalopathy patients [16]. However, the underlying mechanism remains unclear.

Liver is a major regulator of body iron metabolism. Liver dysfunction could disturb iron homeostasis. Yonal *et al* reported elevated serum transferrin saturation and ferritin concentration in patients with hepatitis B virus (HBV)-related liver disease [17]. Matinelli *et al* found elevated transferrin saturation in 27.1% of investigated HBV patients and increased liver iron deposition in 48.7% of the same cohort [18]. Hepcidin, a liver-produced peptide, is considered as the major control of body iron status. Hepcidn regulates body iron homeostasis, by controlling cellular efflux of iron from enterocytes, hepatocytes, and macrophages. Serum prohepcidin, the precursor protein of hepcidin, is significantly low in cirrhotic patients [19]. Yonal et al found lower serum prohepcidin level and its relation with serum ferritin level in HBV-related cirrhotic patients [17]. Based on the evidence that brain iron content could be influenced by systemic iron status [20], we supposed that HBV-related cirrhotic patients had excessive brain iron accumulation, which was influenced by the body iron overload, which could be indicated by serum hepcidin levels.

With the new MRI technique, susceptibility weighted (SW) phase imaging; brain iron could be sensitively detected in vivo [21]. Phase data, derived from the corrected-phase imaging, has been proved to be in a strongly correlation with putative brain iron content in healthy subjects [22]. Thus, we used phase data to quantify brain iron content in our studied population. We further explored the relationship between brain iron deposition and serum hepcidin level, as well as systemic iron status in cirrhotic patients. Herein, we present the evidence of increased regional brain iron deposition in HBV-related cirrhotic patients, and its correlation with serum hepcidin level.

## Patients and Methods

### Ethics Statement

The protocol was approved by ethic committee of Zhongshan Hospital Affiliated to Fudan University (local ethics committee). Written informed consent was obtained from all the participants before the study. All procedures are in accordance with the Helsinki Declaration of 1975.

### Subjects

We enrolled 70 patients with HBV-related cirrhosis, who referred to the clinic of liver diseases between July, 2009 and December, 2010. The inclusion criteria were: (1) age between 30 and 75 years; (2) cirrhosis was proven histologically and related to chronic HBV infection according to the positive serum test for HBV surface antigen (HBsAg); (3) HBV-DNA replication was lower than 1*10ˆ5 copies. The exclusion criteria were: (1) patients with other causes of cirrhosis, such as hepatitis C virus (HCV) infection, alcoholic assumption, Wilson’s disease, hereditary hemochromatosis, autoimmune hepatitis, and primary biliary cirrhosis; (2) patients with abnormal results of high-sensitivity C-reactive protein (hs-CRP), blood routine, renal function, and electrocardiogram, which suggested co-morbid disorders, such as acute inflammatory diseases, hematological disorders, renal failure and heart disease; (3) patients with history of neurological disorders or psychiatric problems; (4) patients who had transfusion or blood loss (>200 ml) in the 2 months prior to the study; (5) patients who had abnormal brain T1 hyperintensity lesions on routine MRI scan.

Forty age- and gender- matched healthy volunteers, who attended a health check-up in ZhongShan Hospital during the study period, served as controls. All controls were judged to be in good health, with normal results on liver function tests and no history of liver diseases.

To avoid the potential bias, we asked every patient and healthy control to complete a food questionnaire for each meal within 4 days before blood test. Dietary iron intake was assessed as previous reported [23]. Individuals who over intake iron based on dietary iron bio-availability were excluded from measurements of serum hepcidin and serum iron-related parameters. (According to Food and Agriculture Organization of the United Nations (FAO), male adolescents with iron intake above 27.4 mg/day and post-menopausal females with iron intake above 22.6 mg/day were excluded.).

### Laboratory Evaluation

After an overnight fast, 4 ml venous blood sample was collected into serum tube, and was centrifuged at 3000 rpm for 10 min. Screening tests, such as liver function, full blood count, were routinely performed at the clinic for every subject. After centrifugation, serum was stored at −80°C for the measurements of serum iron-related parameters and high-sensitivity C-reactive protein. Total iron binding capacity (TIBC) was calculated from the sum of serum iron and unsaturated iron-binding capacity (UIBC), both of which were determined by direct colorimetric methods (Wako Pure Chemical Industries, Osaka, Japan). Serum ferritin level was measured by electrochemiluminescence immunoassay (ECLIA) technology (Roche Diagnostic, Mannheim, Germany). Soluble transferrin receptor (sTfR) and serum transferrin were evaluated by immunoturbidimetric assay (Siemens Healthcare Diagnostics Products GmbH, Marburg, Germany; Roche Diagnostic, Mannheim, Germany ). Transferrin saturation was calculated and expressed as a percentage (serum iron/TIBC ×100%). Serum hepcidin was measured by enzyme-linked immunosorbent assay (ELISA) (DRG Instruments GmbH, Marburg, Germany). High-sensitivity C-reactive protein was also assessed by immunoturbidimetric assays (DiaSys Diagnostic Systems GmbH, Holzheim, Germany). All levels were quantified following the manufacturer’s instructions. Determinations were performed in duplicate. As for the definition of anemia, it was determined as hemoglobin level less than 130 g/L in male adolescents and less than 115 g/L is in females [24]. The reference range for hs-CRP in our lab was 0–3 mg/L, and for albumin was 35 g/L–45 g/L. A serum albumin level of 35 g/L was used as the cut-off point in cirrhotic patients.

### MRI Procedure

MRI was performed on a 3.0 T clinical scanner with an eight-channel phased array head coil (Signa HDx, GE Medical System, Milwaukee, WI). The head was immobilized in the head coil with foam padding. Sagittal T1-weighted images were first acquired to locate the precise positions of the anterior and posterior commissures. We measured conventional axial T2-weighted fast spin-echo [repetition time (TR) = 3400 ms, echo time (TE) = 110 ms, field of view (FOV) = 240 mm, matrix size = 320×256, slice thickness = 5 mm, number of excitations (NEX) = 1] and T1-weighted spin-echo [repetition time (TR) = 1750 ms, echo time (TE) = 24 ms, field of view (FOV) = 240 mm, matrix size = 320×256, slice thickness = 5 mm, number of excitations (NEX) = 1] imaging sequences. All planar sequence acquisitions were obtained in the plane of the anterior commissure–posterior commissure line.

Susceptibility weighted phase images were taken parallel to the anterior–posterior commissural line (AC–PC line) using a three-dimensional gradient-echo sequence with the following parameters: TR = 500 ms, TE = 20 ms, flip angle = 20°, Nz = 36 slices, slice thickness = 2 mm, FOV = 24 cm and matrix size (Nx×Ny) = 256×256. Both phase and magnitude images were acquired, but only phase data were used for further analysis.

### Image Processing and Data Acquisition

The susceptibility weighted phase image was filtered with a high-pass filter to create a new phase map termed the corrected phase image (CPI) [25]. The phase values of the regions of interest (ROIs) were measured on the CPIs. The ROIs included the bilateral globus pallidus, putamen, caudate, thalamus, substantia nigra, red nucleus and frontal white matter. The ROIs of the subcortical nuclei were drawn according to the anatomical structures, while, in the frontal white matter, the ROIs were circular (100 pixels). Phase values of the frontal basal ganglia-thalamocortical circuits were acquired. Datas for each structure were obtained from three contiguous sections, including the section in which the ROI was the largest and its adjacent two sections superiorly and inferiorly. A trained neuroradiologist who was blinded to the age and gender of the subjects, manually traced the ROIs (Fig. S1 in File S1). All the ROIs were re-measured one month later by the same person on the same images. Intraclass correlation coefficient (ICC) was calculated to check the repeatability (Table S1 in File S1). The final value was the means of these two measurements.

To verify our data, we correlated the phase values, for the bilateral globus pallidus, putamen, caudate, thalamus, substantia nigra, red nucleus and frontal white matter in healthy controls, with the postmorterm brain iron concentrations reported by Hallgren and Sourander [26]. We also calculated a formula to depict the relation between phase values and brain iron concentrations in healthy controls. By this formula, we transformed phase values to brain iron concentrations in the ROIs, which were used in correlation analysis between systemic iron overload and brain iron accumulation in patients.

### Statistics

Statistical analysis was performed using SPSS 16.0. The Kolmogorov-Smirnov test was used to verify the normality of distribution of continuous variables. Data was presented as mean values ± SD (range), or as count (percentage), if appropriate. Categorical variables were analyzed using the chi-square test. Intraclass correlation coefficient (ICC) was calculated using a two-way consistency model to estimate measurement error between two measurements. Correlation between quantitative variables was assessed using Pearson correlation coefficient. Because of the large variance of serum ferritin, we use logarithm (base 10) of serum ferritin instead in the correlation analysis. For continuous variables, the difference between two groups was evaluated with an independent-sample t test or the Mann-Whitney U test according to the variable distribution. Analysis of variance (ANOVA) or Kruskal-Wallis test was used in three group comparisons. A value of P<0.05 (two sided) was considered as statistical significance.

## Results

### Baseline Characteristics of Study Population

The demographics and serological data of the study population were summarized in Table 1. All participants were right-handed. In 70 patients, the average value of serum albumin was 35.39±6.05 g/L (22–48 g/L), 25 (35.71%) of which had serum albumin level lower than 35 g/L. Additionally, the mean value of hemoglobin in male patients was 126.20±23.82 g/L (66–169g/L),which was significantly lower than male controls (159.86±12.80 g/L). In healthy controls, levels of serum albumin and hemoglobin were within normal range.

**Table 1:** The demographics and biochemical parameters of cirrhotic patients and healthy controls.

	Cirrhosis Patients (n = 70)	Healthy Controls (n = 40)	*P*-values
Age (years)	49.52±7.91 (31–62)	49.58±7.91 (37–70)	NS
Sex: male/female (n)	66/4	35/5	NS
Child-Pugh classification A/B/C (n)	39/24/7	NA	
Child-Pugh Score (range)	6.6±1.91 (5–12)	5	
Male* Hemoglobin (g/L) (range)	126.20±23.82 (66–169 g/L)	159.86±12.80 (136–189 g/L)	<0.001
Female** Hemoglobin (g/L) (range)	113.00±20.56 (96–140 g/L)	136.00±8.00 (128–144 g/L)	NS
Serum albumin (g/L) (range)	35.39±6.05 (22–48 g/L)	48.86±3.16 (44–55 g/L)	<0.001
hs-CRP	11.56±15.4	1.6±0.99	*<*0.01

NA: not applicable; NS: not significant; hs-CRP: high-sensitivity C-reactive protein.

* Cirrhosis Patients, n = 66; healthy Controls, n = 35.

** Cirrhosis Patients, n = 4; healthy Controls, n = 5.

At the inclusion, five patients had previous episodes of gastrointestinal (GI) bleeding, but none of 70 patients had active GI bleeding or overt hepatic encephalopathy during the study period.

### Correlations between Phase Values and Brain Iron Concentrations of Healthy Controls

The age-related increase in iron level in the basal ganglia is well described in postmortern study [26] and in MR in vivo study [27]. Otherwise, the basal ganglia were the brain regions where excessive iron accumulated in hereditary haemochromatosis, a disease characterized by excessive parenchyma iron deposition [28]. Thus, our study focused on the basal ganglia.

**Figure 1:**
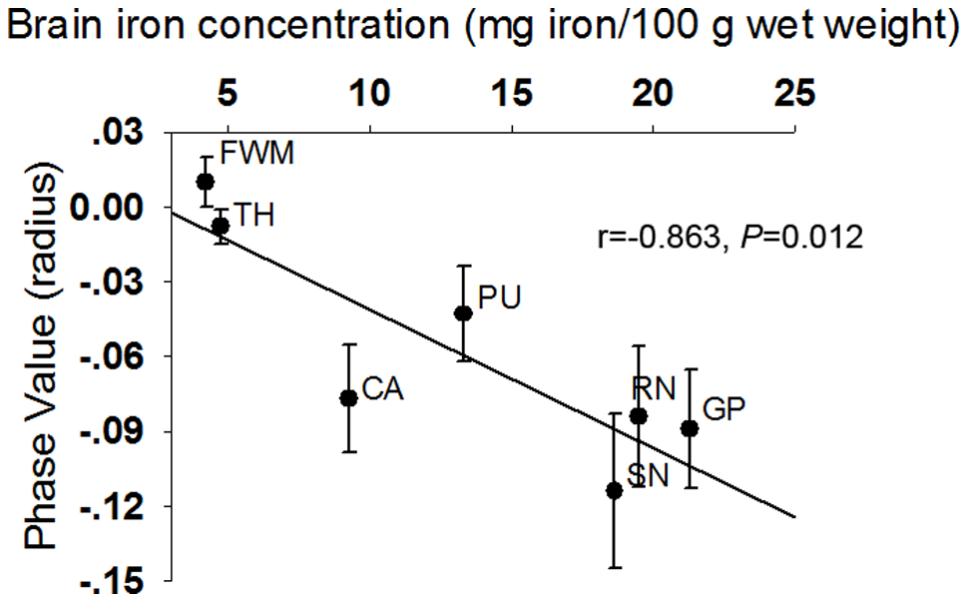
Correlation of phase value with postmortem iron concentrations in brain subregions in 40 healthy controls. Plots of bilateral average phase values for seven brain regions in healthy controls. The linear regression fit to the data was shown by the solid line. RN, red nucleus; SN, substantia nigra; TH, thalamus; CA, caudate; PU, putamen; GP, globus pallidus; FWM, frontal white matter.

Figure 1 presented the correlation between phase values of our healthy controls and postmortem iron concentrations in the bilateral subregions reported by Hallgren and Sourander [26]. Our phase values significantly correlated with the reported brain iron concentrations (r = −0.863; *P* = 0.012) in a linear manner. The formula was: y = −0.0055x+0.0142, where y was the phase value, and x was the brain iron concentration.

### Cirrhosis Patients had Increased Brain Iron Accumulation Compared to Healthy Controls

**Figure 2:**
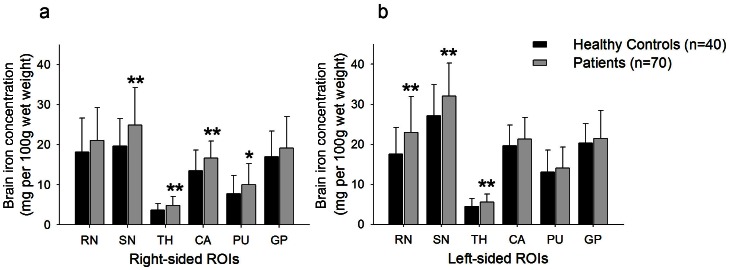
Comparison of brain iron concentration between healthy controls and cirrhosis patients. Bar plots of the average iron concentration with standard deviations for each ROIs of (a) the right and (b) the left hemisphere in healthy controls and patients with cirrhosis. *P<0.05; **P<0.01 (difference of phase value in comparison to healthy control group was evaluated with an independent-sample t test) RN, red nucleus; SN, substantia nigra; TH, thalamus; CA, caudate; PU, putamen; GP, globus pallidus; ROI, region of interest.

We measured phase values for the bilateral red nucleus, substantia nigra, caudate, putamen, globus pallidus, and thalamus (Table S2 in File S1) and calculated brain iron concentration with the formula mentioned above. We found cirrhotic patients had significant increased iron concentrations (mg per 100g wet weight) in the left red nucleus (22.95±9.01 *vs.* 17.58±6.64, *P* = 0.001), the bilateral substantia nigra (right, 24.90±9.36 *vs.* 19.68±6.86, *P* = 0.001; left, 32.06±8.22 *vs.* 27.09±7.81, *P* = 0.002), the bilateral thalamus (right, 4.83±2.28 *vs.* 3.70±1.56, *P* = 0.007; left, 5.54±1.98 *vs.* 4.44±2.00, *P* = 0.006), the right caudate (16.71±4.17 *vs.* 13.49±5.17, *P* = 0.001 ), and the right putamen (10.05±5.22 *vs.* 7.79±4.53, *P* = 0.024), when compared to healthy controls (Fig. 2).

**Figure 3:**
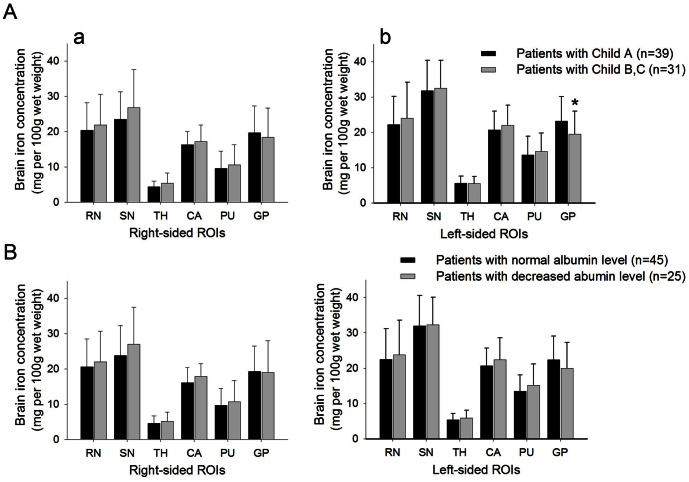
Comparison of brain iron concentration between subgroups of patients by liver function. **A**. Bar plots of the average iron concentration with standard deviations for each ROIs of (a) the right and (b) the left hemisphere in patients with different grade of Child-Pugh classification. **B**. Bar plots of the average iron concentration for each ROIs in cirrhosis patients divided by their serum albumin level. *P<0.05 (in comparison with Child B, C patients evaluated with an independent-sample t test).

To explore whether brain iron accumulated as liver dysfunction exacerbated in cirrhotic patients, we further compared brain iron concentrations between patients with different degree of liver dysfunction. The severity of liver dysfunction was evaluated by either Child-Pugh classification or serum albumin level. Patients with Child-Pugh Class A had increased brain iron accumulation in the left globus pallidus than Class B and C patients (23.13±6.92 *vs.* 19.46±6.51, *P* = 0.027) (Fig. 3a). When divided by serum albumin level, patients of two subgroups did not have significantly different iron concentrations in ROIs. (Fig. 3b).

### HBV-related Cirrhotic Patients had Decreased Serum Hepcidin Level and Increased Body Iron Status

In order to study the body iron status of cirrhotic patients accurately, we tested hepcidin and iron-related serum parameters in 30 patients, and 19 age- and gender-matched healthy controls. These patients and controls didn’t over intake iron based on dietary iron bio-availability before blood tests. The phase values were decreased in patients compared to those of controls (Table S3 in File S1). Brain iron concentrations were significantly increased in the bilateral caudate, red nucleus and the right substantia nigra of cirrhotic patients compared to those of healthy controls (Fig. S2 in File S1).

We measured serum bioactive hepcidin concentrations in patients and healthy controls, to explore whether body iron overload was due to iron dysregulation by hepatocytes. Cirrhotic patients had significantly lower serum hepcidin level than healthy controls (18.31±9.67 ng/mL *vs.* 28.87±7.41 ng/mL, *P* = 0.000), which indicated potential disturbed body iron regulation in cirrhotic patients (Table 2).

**Table 2:** The baseline characteristics and laboratory tests of hepcidin and body iron status in cirrhotic patients and healthy controls.

	Cirrhosis Patients (n = 30)	Healthy Controls (n = 19)	*P*-values
Age (years)	50.37±7.78 (33–62)	49.74±8.88 (38–70)	NS
Sex: male/female (n)	29/1	17/2	NS
Hemoglobin (g/L)	125.40±20.92	151.83±10.48	<0.01
Serum albumin (g/L)	34.2±6.12	46.67±2.73	<0.001
Serum iron (umol/L)	19.41±9.60	19.02±7.59	NS
Transferrin (g/L)	1.77±0.50	2.56±0.39	<0.001
TIBC (umol/L)	36.53±10.20	53.25±6.78	<0.001
Transferrin Saturation (%)	53.60±22.93	35.52±13.53	<0.01
Soluble Transferrin Receptor (mg/L)	1.41±0.55	1.21±0.31	NS
Ferritin (ng/mL)	536.26±587.49	308.58±244.49	NS
Hepcidin (ng/mL)	18.31±9.67	28.87±7.41	<0.001

TIBC: total iron binding capacity; NS: not significant.

The values of serum iron parameters were shown in Table 2. Cirrhotic patients had significantly decreased serum transferrin (*P* = 0.000), TIBC (*P* = 0.000), and elevated transferrin saturation (*P* = 0.002), when compared with healthy controls. Patients had higher serum ferritin concentration and soluble transferring receptor than healthy controls did (serum ferritin, 536.26±587.49 ng/mL *vs.* 308.58±244.49 ng/mL; sTfR, 1.41±0.55 mg/L *vs.* 1.21±0.31 mg/L). Nevertheless, the differences did not reach the statistical significance.

We found significantly decreased hepcidin concentration, combined with increased transferrin saturation (greater than 50%) and high level of serum ferritin (greater than 500 ng/mL) in cirrhotic patients, which indicated body iron overload. Our results were consistent with previous researches [17], [18], [29], [30].

### Serum Hepcidin was Correlated with Regional Brain Iron Concentration in HBV-related Cirrhotic Patients

**Figure 4:**
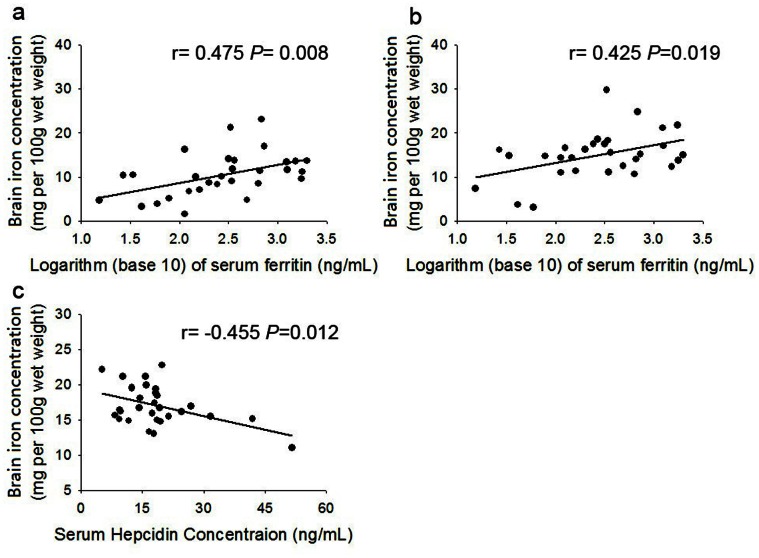
Correlation of log ferritin or serum hepcidin concentration with regional brain iron concentration in cirrhosis. **A.** Plots of the average iron concentration in the right PU versus logarithm (base 10) of serum ferritin in cirrhosis patients. The linear regression fit to the data was shown by the solid line. **B.** Plots of the average iron concentration in the left PU versus logarithm (base 10) of serum ferritin in cirrhosis patients. The linear regression fit to the data was shown by the solid line. **C.** Plots of the average iron concentration in the right CA versus serum hepcidin concentration in cirrhotic patients. The linear regression fit to the data was shown by the solid line.

We further performed Pearson correlation analysis to explore the relation of brain iron concentration with serum hepcidin level in cirrhotic patients. The iron concentration in each ROI was calculated by the formula mentioned above. We found that serum hepcidin level was in a negative relationship with brain iron concentration in the right caudate (r = −0.455, *P* = 0.012). We also found logarithm of serum ferritin positively correlated with brain iron concentration of the right putamen (r = 0.475, *P* = 0.008), and the left putamen (r = 0.425, *P* = 0.019). (Fig. 4).

## Discussion

In this study, we found increased brain iron deposition in HBV-related cirrhotic patients, using SW corrected-phase imaging. We reported decreased serum hepcidin level in the cirrhotic patients with body iron overload, characterized by decreased TIBC, transferring, and increased transferring saturation. Furthermore, we found excessive brain iron deposition in patients significantly correlated with decreased serum hepcidin level and increased serum ferritin level. These significant correlations support our assumption that the abnormal brain iron accumulation in HBV-related cirrhotic patients may due to systemic iron overload.

In chronic virus hepatitis, disturbed iron homeostasis and body iron overload are common [30]. But only Eng and his colleagues reported excessive brain iron accumulation in three patients with hepatic iron overload, due to end-stage liver disease [31]. We calculated brain iron concentration in the basal ganglia to estimate the extent of brain iron deposition. We presented the convincing evidence that HBV- related cirrhotic patients had brain iron repletion in the left red nucleus, the bilateral substantia nigra, the bilateral thalamus, the right caudate, and the right putamen, by susceptibility weighted phase imaging.

Susceptibility weighted phase imaging is a technique sensitive for iron [32], [33]. Post-mortem studies showed a strong linear correlation between phase value and chemically determined brain iron concentration [34], [35]. Susceptibility weighted phase imaging had been used to quantify brain iron content in healthy population [22], [27] and in patients with Parkinson’s disease, Alzheimer’s disease or multiple sclerosis [25], [36], [37]. We reported that patients with HBV-related hepatic cirrhosis had excessive brain iron accumulation in the basal ganglia, which was consistent with our previous research [16].

Body iron overload was suspected to be responsible for increased brain iron accumulation. House et al found high R_2_ (proton transverse relaxation rate) values in gray matter regions positively correlated with liver iron concentration and serum transferrin saturation, which indicated brain iron level could be influenced by systemic iron status [20]. Corengia *et al* reported patients with hepatitis C infection had excessive body iron accumulation [38]. Patients with HBV hepatitis were also found to have abnormal serum iron parameters. The serum levels of iron and ferritin are elevated in up to 36% and 30%, respectively, of patients with chronic hepatitis including HBV infection [29]. Elevated transferrin saturation was found in 27.1% patients with chronic HBV infection [18].

In our study, we found decreased serum transferrin and TIBC, as well as increased transferrin saturation in HBV-related cirrhotic patients compared with controls, which was consistent with other studies [18], [29]. We observed a higher level of serum ferritin in cirrhotic patients, as found by Yonal [17], [30]. Our result also indicated that the increased serum ferritin was positively correlated with excessive brain iron accumulation in the bilateral putamen.

Our results further demonstrated that serum hepcidin was much lower in HBV-related cirrhotic patients than in controls. Hepcidin is an acute phase reactant protein that is produced and secreted predominantly by hepatocytes. It is widely accepted that the liver regulates body iron via hepcidin. Hepcidin negatively regulates the egress of iron from cells by binding to the cellular iron exporter ferroportin and inducing its internalization and degradation [39], [40], [41]. However, due to the previous absence of an accurate serum assay, most studies of hepcidin in human measure the serum level of prohepcidin, the peptide precursor of hepcidin, or urine hepcidin [42], [43]. These studies are difficult to interpret because the relationship between hepcidin, prohepcidin, and urine hepcidin remains unclear. In our study, we are the first to report the quantitative measurements of bioactive serum hepcidin in HBV-related cirrhotic patients. We found significantly decreased serum hepcidin in cirrhotic patients. This was consistent with study which reported the decreased expression of hepcidin mRNA in chronic hepatitis C [44]. The decreased hepcidin concentration results in the release of stored iron and in an increase in the dietary iron absorption, which cause body iron overload in cirrhotic patients [45]. We also found the decreased serum hepcidin concentration was in a close relationship with increased brain iron deposition in the right caudate in cirrhotic patients. Hence, this result indicated that brain iron repletion, in HBV-related cirrhosis patients, may be influenced by systemic iron dysregulation by hepatocytes. Therefore, based on these findings in HBV-related cirrhosis patients we supposed increased brain iron accumulation may relate to the body iron overload.

Chronic normochromic, normocytic or macrocytic anaemia is a common feature of cirrhosis [46]. Our research also showed significant low hemoglobin levels in patients compared to controls. The reason of anemia in cirrhosis may be multifactorial, including decreased erythrocyte survival, reduced erythropoietin levels or hypersplenism [47]. Hepcidin could be suppressed by anemia. The exact molecular pathway is still unclear. Anemia initiates a cascade of adaptive responses aimed at boosting erythropoiesis. Previous research found that when hematopoiesis was inhibited, hepcidin expression increased despite severe anemia. It suggested that hepcidin is exclusively sensitive to iron utilization for erythropoiesis [48]. sTfR has long been established as an index of erythropoiesis [49]. It reflects the number of transferrin receptors on immatured red cells, which presents the level of bone marrow erythropoiesis. Importantly, sTfR does not elevate in hepatitis, or chronic liver disease [50]. Thus, sTfR is considered a good serum marker of erythropoietic activity in patients with liver disease. We have measured levels of sTfR and found that sTfR values were not significantly elevated in patients compared to those of controls. Our finding indicated that bone marrow erythropoiesis was not over activated in our patients. Thus, anemia-driven erythropoiesis has little influence on serum hepcidin level in our patients.

To explore whether brain iron extensively accumulated as liver dysfunction exacerbated, we compared brain iron concentration between patients divided by their degrees of liver dysfunction. However, we did not find convincing trends for brain iron deposition when liver dysfunction was graded either by Child-Pugh classification or serum albumin level.

The pathological mechanisms by which brain iron accumulates in liver diseases are not thoroughly explained [18]. Based on our results, we assumed that the increased brain iron concentration was due to over-activated iron transportation into brain, induced by the systemic iron overload in cirrhotic patients. Systemic iron status dictated the regulation of brain iron uptake by influencing the endothelia cell of brain-blood barrier (BBB) [51], where transferrin/transferrin receptor (Tf/TfR) pathway was the major route [52]. In pathological body iron overload, non-transferrin bound iron (NTBI) also participated in the transportation of iron across the BBB, when Tf was saturated [51]. After the iron was transported across the BBB, it was mainly bound to transferrin and was transported within the brain. The mechanism of the preferential iron deposition in the basal ganglia is unknown. Some postulated the pattern of pathological iron accumulation may follow that of normal brain iron distribution [53], which highest iron content was in the basal ganglia proven by both postmortem and in vivo study [26]. The excessive iron in the basal ganglia catalyzed reactions forming oxygen radicals, which stimulated damage to the mitochondrial electron transport, induction of proteases and increase membrane lipid peroxidation [54]. This may partially explain the neurological symptoms found in cirrhotic patients.

There were some limitations in our study. Our cross-sectional study could not depict a clear cause-effect relationship between increased brain iron accumulation and neurological manifestations in cirrhosis patients. The relative small population may also hinder a better interpretation of our results. Our preliminary results merit further investigations.

In conclusion, we have shown patients with HBV-related cirrhosis had increased brain iron accumulation in the basal ganglia, as well as decreased serum hepcidin which was in close relationship with the increased brain iron accumulation in the right caudate. As a key modulator of systemic iron metabolism, serum hepcidin may be a potential biomarker for brain iron deposition in cirrhotic patients.

## Supporting Information

File S1 **Contains: Figure S1 Illustration of the bilateral regions of interest on the corrected phase images in one cirrhotic patient.** CA, caudate; FWM, frontal white matter; GP, globus pallidus; PU, putamen; RN, red nucleus; SN, substantia nigra; TH, thalamus. **Figure S2 Comparison of average iron content in brain subregions between 19 healthy controls and 30 cirrhosis patients.** Plot - error bar of the average iron concentration with standard deviations for each ROIs of (a) the right and (b) the left hemisphere in nineteen healthy controls and thirty cirrhotic patients. * = significant difference of iron content between the patients and control groups. (*P*<0.05, two-tailed t test); ** = significant difference of average iron concentration between the patients and control groups (*P*<0.01, two-tailed t test). CA, caudate; GP, globus pallidus; PU, putamen; RN, red nucleus; ROI, region of interest; SN, substantia nigra; TH, thalamus. **Table S1 Measurement of the inter-measure differences between mean values of bilateral average phase values in subjects with HBV-related cirrhosis.** CA, caudate; FWM, frontal white matter; GP, globus pallidus; ICC, intraclass correlation coefficients; PU, putamen; RN, red nucleus; SN, substantia nigra; TH, thalamus. **Table S2 Phase values of regions of interest in 70 cirrhotic patients and 40 healthy controls.** CA, caudate; FWM, frontal white matter; GP, globus pallidus; PU, putamen; RN, red nucleus; SN, substantia nigra; TH, thalamus. **Table S3 Phase value of regions of interest in 30 cirrhotic patients and 19 healthy controls.** CA, caudate; FWM, frontal white matter; GP, globus pallidus; PU, putamen; RN, red nucleus; SN, substantia nigra; TH, thalamus.
(DOC)
