# Supplementary material for: Decreased Serum Hepcidin Concentration Correlates with Brain Iron Deposition in Patients with HBV-Related Cirrhosis
Source: PLoS One. 2013 Jun 11;8(6):e65551. doi: 10.1371/journal.pone.0065551 (PMC3679136; doi:10.1371/journal.pone.0065551)
Supplement: File S1 — Contains: Figure S1 Illustration of the bilateral regions of interest on the corrected phase images in one cirrhotic patient. CA, caudate; FWM, frontal white matter; GP, globus pallidus; PU, putamen; RN, red nucleus; SN, substantia nigra; TH, thalamus. Figure S2 Comparison of average iron content in brain subregions between 19 healthy controls and 30 cirrhosis patients. Plot - error bar of the average iron concentration with standard deviations for each ROIs of (a) the right and (b) the left hemisphere in nineteen healthy controls and thirty cirrhotic patients. * = significant difference of iron content between the patients and control groups. (P<0.05, two-tailed t test); ** = significant difference of average iron concentration between the patients and control groups (P<0.01, two-tailed t test). CA, caudate; GP, globus pallidus; PU, putamen; RN, red nucleus; ROI, region of interest; SN, substantia nigra; TH, thalamus. Table S1 Measurement of the inter-measure differences between mean values of bilateral average phase values in subjects with HBV-related cirrhosis. CA, caudate; FWM, frontal white matter; GP, globus pallidus; ICC, intraclass correlation coefficients; PU, putamen; RN, red nucleus; SN, substantia nigra; TH, thalamus. Table S2 Phase values of regions of interest in 70 cirrhotic patients and 40 healthy controls. CA, caudate; FWM, frontal white matter; GP, globus pallidus; PU, putamen; RN, red nucleus; SN, substantia nigra; TH, thalamus. Table S3 Phase value of regions of interest in 30 cirrhotic patients and 19 healthy controls. CA, caudate; FWM, frontal white matter; GP, globus pallidus; PU, putamen; RN, red nucleus; SN, substantia nigra; TH, thalamus. (DOC) [file pone.0065551.s001.doc]

**Supporting Information**

**The high reliability of two measurements of the regions of interest on the corrected phase image**

To assess the consistency between two quantitative measurements, we evaluated the reliability throught the intraclass correlation coefficients (ICC). We calculated the ICC using a two-way consistency model to estimate the measurement error. We found the high value of ICC, which meant a high agreement between two measurements. (Table S1)

**Higher brain iron level in the bilateral red nucleus, the right substantia nigra, and the bilateral caudate in 30 cirrhotic patients than 19 healthy controls**

We compared brain iron concentrations in ROIs between 30 cirrhotic patients and 19 healthy controls, all of whom had tests of serum iron parameters and hepcidin. We found cirrhotic patients had decreased brain iron content in the bilateral red nucleus (right, 22.32 ± 8.48 vs. 17.46 ± 4.67, P < 0.05; left, 24.08 ± 9.49 vs. 18.13 ± 6.86, P < 0.05), the right substantia nigra (26.09 ± 11.51 vs. 20.21 ± 6.57, P < 0.05), and the bilateral caudate (right, 17.22± 2.79 vs. 12.36 ± 5.67 P < 0.01; left, 21.94 ± 5.91 vs. 18.15 ± 5.45, P<0.05). (Figure S2)

Phase values of all ROIs in patients and controls were demonstrated in Table S2. Phase values of all ROIs in patients and controls that had tests of serum iron parameters and hepcidin were demonstrated in Table S3.

**Supporting Information Legends**

**Figure S1 Illustration of the bilateral regions of interest on the corrected phase images in one cirrhotic patient**

CA, caudate; FWM, frontal white matter; GP, globus pallidus; PU, putamen; RN, red nucleus; SN, substantia nigra; TH, thalamus.

**Figure S2. Comparison of average iron content in brain subregions between 19 healthy controls and 30 cirrhosis patients**

Plot - error bar of the average iron concentration with standard deviations for each ROIs of (a) the right and (b) the left hemisphere in nineteen healthy controls and thirty cirrhotic patients. *=significant difference of iron content between the patients and control groups.(*P* < 0.05, two-tailed t test); **=significant difference of average iron concentration between the patients and control groups (*P*<0.01, two-tailed t test).

CA, caudate; GP, globus pallidus; PU, putamen; RN, red nucleus; ROI, region of interest; SN, substantia nigra; TH, thalamus.

**Table S1 Measurement of the inter-measure differences between mean values of bilateral average phase values in subjects with HBV-related cirrhosis**

|  | **Phase 1^st^ Measurement (radians)** | **Phase 2^nd^ Measurement (radians)** | **Mean difference** | **ICC** |
| --- | --- | --- | --- | --- |
| **RN** | -0.111 ± 0.043 | -0.113 ± 0.048 | 0.002 | 0.972 |
| **SN** | -0.137 ± 0.043 | -0.144 ± 0.049 | 0.007 | 0.964 |
| **TH** | -0.014 ± 0.011 | -0.013 ± 0.010 | 0.000 | 0.903 |
| **CA** | -0.098 ± 0.019 | -0.093 ± 0.020 | 0.005 | 0.947 |
| **PU** | -0.056 ± 0.27 | -0.056 ± 0.027 | 0.000 | 0.985 |
| **GP** | -0.083 ± 0.012 | -0.087 ± 0.031 | 0.003 | 0.874 |
| **FWM** | 0.0015 ± 0.011 | -0.00049 ± 0.013 | 0.002 | 0.978 |

CA, caudate; FWM, frontal white matter; GP, globus pallidus; ICC, intraclass correlation coefficients; PU, putamen; RN, red nucleus; SN, substantia nigra; TH, thalamus.

**Table S2** Phase values of regions of interest in 70 cirrhotic patients and 40 healthy controls

|  |  | **HBV-related Cirrhosis Patients (n=70)** | **Healthy Controls (n=40)** | ***P* - values** |
| --- | --- | --- | --- | --- |
| **RN** | Right | -0.102 ± 0.045 | -0.086 ± 0.047 | 0.08 |
|  | Left | -0.112 ± 0.050 | -0.082 ± 0.037 | 0.001 |
| **SN** | Right | -0.122 ± 0.051 | -0.094 ± 0.038 | 0.001 |
|  | Left | -0.162 ± 0.045 | -0.135 ± 0.043 | 0.002 |
| **TH** | Right | -0.012 ± 0.013 | -0.006 ± 0.009 | 0.007 |
|  | Left | -0.016 ± 0.011 | -0.010 ± 0.011 | 0.006 |
| **CA** | Right | -0.078 ± 0.023 | -0.060 ± 0.028 | 0.001 |
|  | Left | -0.102 ± 0.030 | -0.093 ± 0.028 | 0.117 |
| **PU** | Right | -0.041 ± 0.029 | -0.029 ± 0.025 | 0.024 |
|  | Left | -0.063 ± 0.029 | -0.058 ± 0.030 | 0.339 |
| **GP** | Right | -0.091 ± 0.043 | -0.080 ± 0.035 | 0.153 |
|  | Left | -0.104 ± 0.038 | -0.098 ± 0.026 | 0.341 |
| **FWM** | Right | 0.025 ± 0.014 | 0.030 ± 0.018 | 0.171 |
|  | Left | -0.025 ± 0.022 | -0.010 ± 0.009 | 0.000 |

CA, caudate; FWM, frontal white matter; GP, globus pallidus; PU, putamen; RN, red nucleus; SN, substantia nigra; TH, thalamus.

**Table S3** Phase value of regions of interest in 30 cirrhotic patients and 19 healthy controls

|  |  | **HBV-related Cirrhosis Patients (n=30)** | **Healthy Controls (n=19)** | ***P* - values** |
| --- | --- | --- | --- | --- |
| **RN** | Right | -0.109 ± 0.047 | -0.082 ± 0.026 | 0.013 |
|  | Left | -0.118 ± 0.052 | -0.086 ± 0.038 | 0.022 |
| **SN** | Right | -0.129 ± 0.063 | -0.097 ± 0.036 | 0.028 |
|  | Left | -0.160 ± 0.048 | -0.139 ± 0.040 | 0.125 |
| **TH** | Right | -0.0104 ± 0.010 | -0.008 ± 0.010 | 0.368 |
|  | Left | -0.017 ± 0.013 | -0.010 ± 0.014 | 0.064 |
| **CA** | Right | -0.081 ± 0.015 | -0.054 ± 0.031 | 0.002 |
|  | Left | -0.106 ± 0.032 | -0.086 ± 0.030 | 0.029 |
| **PU** | Right | -0.044 ± 0.028 | -0.031 ± 0.031 | 0.153 |
|  | Left | -0.069 ± 0.030 | -0.057 ± 0.040 | 0.230 |
| **GP** | Right | -0.081 ± 0.045 | -0.077 ± 0.029 | 0.728 |
|  | Left | -0.091 ± 0.031 | -0.098 ± 0.028 | 0.506 |
| **FWM** | Right | 0.027 ± 0.013 | 0.032 ± 0.021 | 0.329 |
|  | Left | -0.028 ± 0.024 | -0.009 ± 0.009 | 0.000 |

CA, caudate; FWM, frontal white matter; GP, globus pallidus; PU, putamen; RN, red nucleus; SN, substantia nigra; TH, thalamus.

**Figure S1**

**
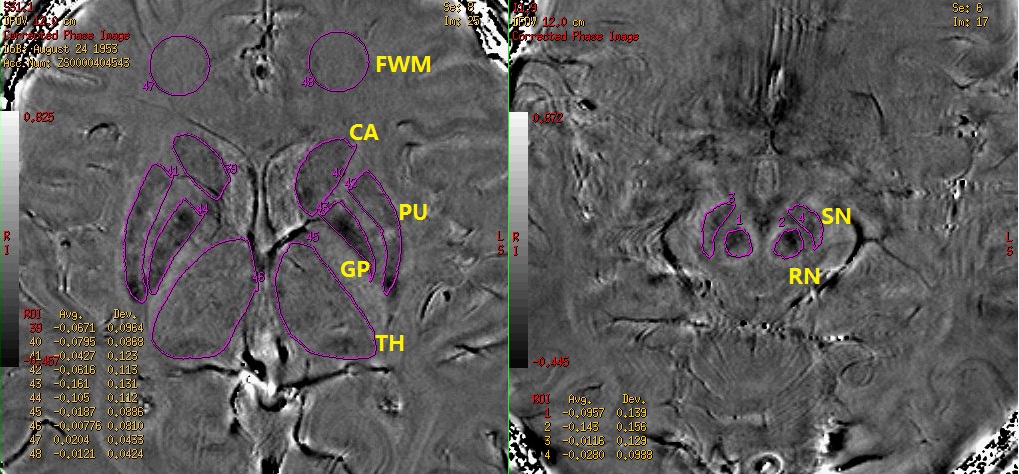
**

CA, caudate; FWM, frontal white matter; GP, globus pallidus; PU, putamen; RN, red nucleus; SN, substantia nigra; TH, thalamus.

**Figure S2**

**
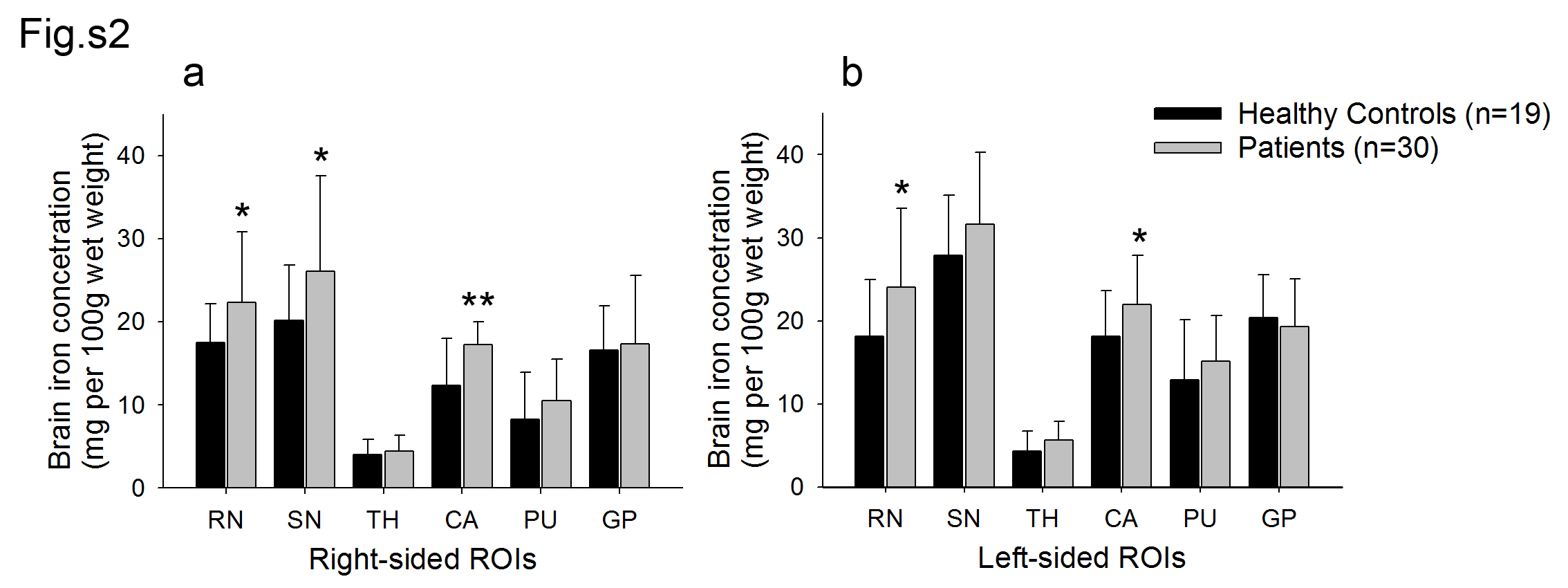
**

CA, caudate; GP, globus pallidus; PU, putamen; RN, red nucleus; ROI, region of interest; SN, substantia nigra; TH, thalamus.
